# Printability of Double Network Alginate-Based Hydrogel for 3D Bio-Printed Complex Structures

**DOI:** 10.3389/fbioe.2022.896166

**Published:** 2022-07-08

**Authors:** Immacolata Greco, Vanja Miskovic, Carolina Varon, Chiara Marraffa, Carlo S. Iorio

**Affiliations:** Université Libre de Bruxelles, Brussels, Belgium

**Keywords:** hydrogels, biomaterials, alginate, shape fidelity, ink viscosity, 3D bio-printing

## Abstract

Three-dimensional (3D) bio-printing has recently emerged as a crucial technology in tissue engineering, yet there are still challenges in selecting materials to obtain good print quality. Therefore, it is essential to study the influence of the chosen material (i.e., bio-ink) and the printing parameters on the final result. The “printability” of a bio-ink indicates its suitability for bio-printing. Hydrogels are a great choice because of their biocompatibility, but their printability is crucial for exploiting their properties and ensuring high printing accuracy. However, the printing settings are seldom addressed when printing hydrogels. In this context, this study explored the printability of double network (DN) hydrogels, from printing lines (1D structures) to lattices (2D structures) and 3D tubular structures, with a focus on printing accuracy. The DN hydrogel has two entangled cross-linked networks and a balanced mechanical performance combining high strength, toughness, and biocompatibility. The combination of poly (ethylene glycol)-diacrylate (PEDGA) and sodium alginate (SA) enables the qualities mentioned earlier to be met, as well as the use of UV to prevent filament collapse under gravity. Critical correlations between the printability and settings, such as velocity and viscosity of the ink, were identified. PEGDA/alginate-based double network hydrogels were explored and prepared, and printing conditions were improved to achieve 3D complex architectures, such as tubular structures. The DN solution ink was found to be unsuitable for extrudability; hence, glycerol was added to enhance the process. Different glycerol concentrations and flow rates were investigated. The solution containing 25% glycerol and a flow rate of 2 mm/s yielded the best printing accuracy. Thanks to these parameters, a line width of 1 mm and an angle printing inaccuracy of less than 1° were achieved, indicating good shape accuracy. Once the optimal parameters were identified, a tubular structure was achieved with a high printing accuracy. This study demonstrated a 3D printing hydrogel structure using a commercial 3D bio-printer (REGEMAT 3D BIO V1) by synchronizing all parameters, serving as a reference for future more complex 3D structures.

## 1 Introduction

Three-dimensional (3D) bio-printing is a manufacturing process that makes it possible to produce heterogeneous objects and high-resolution complex structures with anatomical precision layer by layer using the so-called bio-inks (Ng et al., 2019) (Ashammakhi et al., 2019) (Barrs et al., 2021) (Gu et al., 2020). There are multiple bio-printing technologies, such as those based on extrusion (Suntornnond et al., 2016) (Fu et al., 2021), inkjet-based (Li X. et al., 2020) (Saunders and Derby, 2014), microvalve (Ng et al., 2017a; Ng et al., 2017b), and laser (Lin et al., 2013) (Cerchiari et al., 2019) (Zhang et al., 2020). Extrusion-based bio-printing is the most widely used method due to its quick production time, ease of operation, and compatibility with a variety of bio-inks (Zhuang et al., 2019) (Ng et al., 2022). Therefore, this research focuses on optimizing parameters (e.g., flow speed and material viscosity) for an extrusion bio-printer. Little attention has been paid to this optimization during printing, and to the relationship between printing parameters and both, printing accuracy and shape fidelity. This will be performed using REGEMAT BIO V1 (REGEMAT, Granada, Spain), since this bio-printer has the desired properties, in terms of cost, size, and weight, which are crucial for future space exploration. The printing accuracy, defined as the degree to which the final product matches the reference shape (Fu et al., 2021), and the shape fidelity, identified as the capacity of the printed construct to keep the shape after deposition, can be used to characterize good printed results (Ribeiro et al., 2020) (Gillispie et al., 2020).

Many parameters will significantly influence printability during the extrusion-based bio-printing process, such as the polymerization time of the inks during printing (Fu et al., 2021) and the dependence of the extrusion from the print speed and the gravity influence on the shape fidelity (Murphy et al., 2020).

Making tissue constructs with the desired functional and biomechanical properties from available biomaterials remains challenging. Tissue engineering scaffolding should consider the impact of manufacturing on cell viability (Ashammakhi et al., 2019). Moreover, printed biostructures should have a proper microarchitecture to offer mechanical stability and promote cell ingrowth (Fedorovich et al., 2008). One approach to tackle this involves the combinations of materials that can provide the tissue with mechanical, structural, and geometrical properties (such as the elastic modulus and tensile strength) at the macroscopic level, and the appropriate structural and biochemical environment for cell encapsulation and placement (Murphy et al., 2020).

For completeness, the creation of a complex scaffold is also possible *via* electrospinning (Nair et al., 2004) and freeze-drying (Fereshteh, 2018), while being cost-effective techniques (Li J. et al., 2020). However, these latter do not allow the necessary precise control of each printed layer to obtain highly complex scaffolds (Li J. et al., 2020) and vascular structure (Jafarkhani et al., 2019) as offered by 3D bio-printing. Furthermore, such processes demand a post-cell seeding procedure.

Building a functional tissue involves forming biostructures that can transport nutrients and oxygen to cells (Rouwkema et al., 2008) while assuring the removal of metabolic byproducts from the body (Wang et al., 2014). In 3D bioprinting, this naturally occurring process is very difficult to reproduce if one considers the variables that impact the structures’ formation and the number of phenomena—diffusion, gas exchange, and cell adhesion—involved (Rouwkema et al., 2008) (Zhu and Marchant, 2011).

The first step for successful 3D bioprinting is the choice of the material, which is not always straightforward because of the difficulty in combining the ink viscoelastic properties allowing for a precise printing and the biocompatibility needed for the final application (Fedorovich et al., 2008) (Bellan et al., 2009). Hydrogels have emerged as a biomaterial largely employed as cell-laden materials for bioprinting due to their unique properties, such as biocompatibility and high fluid content (He et al., 2016). Concerning their printability, much attention has been paid to the rheology and viscosity of the ink (Bellan et al., 2009).

Hydrogels are synthesized by different polymerization methods using both chemical and physical cross-linking routes. Chemically cross-linked hydrogels are synthesized through chain-to-growth polymerization, and one of the most commonly used is UV polymerization. On the other hand, physically cross-linked hydrogels are synthesized by ionic interaction (Maitra and Shukla, 2014) (Mishra and Mishra, 2016).

The advantage of synthetic polymers is that they can be customized with specific physical properties, such as mechanical properties, to suit certain applications. However, their use is questioned by their inadequate biocompatibility, toxic degradation products, and the loss of mechanical properties during degradation (Zhu and Marchant, 2011). Natural polymers, on the other hand, possess the biocompatibility properties required for bio-inks (Barrs et al., 2021) (Ashammakhi et al., 2019).

Poly(ethylene glycol)-diacrylate (PEGDA) hydrogels are popular candidates that satisfy most of the aforementioned properties. This is due to their mechanical properties, and their ability to be formed in a fast and controllable manner in the presence of different cell types. However, their mechanical behavior is very dissimilar to biological tissue since they behave mechanically anisotropic, nonlinear, and viscoelastic (Zhu and Marchant, 2011).

Sodium alginate (SA) is one of the most used natural polymers for tissue engineering due to its excellent biocompatibility (Augst et al., 2006) and ability to quickly obtain hydrogels under physiological conditions (Drury et al., 2004). Moreover, SA is a natural polysaccharide extracted from brown algae, and it has been widely used in biomedical engineering owing to its favorable features, such as good biocompatibility and ease of gelation (Augst et al., 2006). Materials for tissue engineering require high mechanical properties and good biocompatibility, and a good example for obtaining such material is the combination of SA and PEGDA in a double network (DN). A DN hydrogel is composed of two different polymer networks: the first is hard and brittle, while the second is soft and ductile (Nonoyama and Gong, 2015) (Branched, 2015). The two entangled cross-linked networks in the DN hydrogel provide balanced qualities that combine high strength and biocompatibility. In this work, the printability is characterized based on the correlation between printing parameters and the printing accuracy on alginate-based material, from printing lines to printing lattice, as performed in the study by Yong et al. (He et al., 2016). The ability to leverage the properties of distinct polymers to suit the requirements of complex constructions is one of the benefits of adopting a double network approach (Chimene et al., 2020) (Ashammakhi et al., 2019).

This study evaluates the printability of DN alginate-based hydrogels in terms of printing accuracy and shape fidelity. Furthermore, it assesses whether the resultant hydrogels meet the requirements of vascular structures. To this end, the ink viscosity and printing settings are optimized using multiple line widths and angle printing. The reason for using such structures is that they are the most challenging structures in 3D bioprinting (He et al., 2016). Once the optimal printing settings are identified, the printing accuracy and the shape fidelity of a tubular structure are evaluated.

## 2 Materials

For this work, sodium alginate (SA), poly(ethylene glycol)-diacrylate (PEDGA) (average Mn700), calcium chloride (CaCl_2_), glycerol 99%, and irgacure 2959 (2-hydroxy-4’-(2-hydroxyethoxy)-2-methylpropiophenone) (I2959) were purchased from Sigma-Aldrich. CaCl_2_ solution was prepared by dissolving CaCl_2_ powder into distillate water to obtain the final concentration of 2.5% (w/v).

### 2.1 Hydrogel Solution Preparation

Four sets of samples were prepared (see Table 1). PEGDA was first dissolved in distilled water at 40% (w/v) concentration, and photoinitiator I2959 was added to the PEGDA precursor solution at 1% (w/v) concentration. After that, a double network solution was obtained, adding 4% (w/v) of SA. Different glycerol concentrations were tested to study the influence of the viscosity on the printing process. All the solutions, before the cross-linking, were stirred until they became homogeneous and degassed for 2 h to eliminate air bubbles. The solutions were then polymerized by ultraviolet (UV wavelength 365 nm) irradiation during the printing process. Then, the obtained hydrogels were transferred into the CaCl_2_ solution for 5 h to achieve the cross-linking of alginate. The purpose of choosing this approach was to avoid the filament from collapsing during the printing process.

**TABLE 1:** Set of prepared samples.

Sample	PEGDA [%]	SA [%]	Glycerol [%]
DN	40	4	-
DN15	40	4	15
DN25	40	4	25
DN35	40	4	35

## 3 Methodology

This section describes the different tests that were carried out to evaluate the properties of the samples. The tested properties are crucial because they allow the definition of the right composition and the optimal settings to bio-print using the REGEMAT 3D BIO V1 printer (REGEMAT, Granada Spain).

### 3.1 Rheological Test

The rheological properties of the prepared mixtures are important to optimize the ink viscosity, thereby preventing the needle occlusion during the printing process. In this study, they were determined using a modular compact rheometer (MCR) 302 (Anton Paar, Belgium) using parallel-plate geometry. The viscosity was measured as a shear rate function at a constant temperature of 25°C, and the shear rate varied from 1 to 100 $s^{-1}$ . The non-Newtonian behavior of DN solutions was modeled using the two-parameter power-law model, which is the most frequently used model for alginate solutions (Basim and Fara, 2013):

$$\eta =K\gamma^{n-1},$$ (1)

where η is the apparent viscosity, K is the consistency coefficient, γ is the shear rate, and n is the flow index.

### 3.2 Hydrogel Bioprinting

For this work, the printing process was carried out using Regemat 3D BIO V1, with the addition of the UV lamp (wavelength 365 nm) for the simultaneous cross-linking process of the PEGDA during printing. Hydrogel solutions were loaded into the syringe, with a needle diameter (D) of 0.41 mm.

**FIGURE 1:**
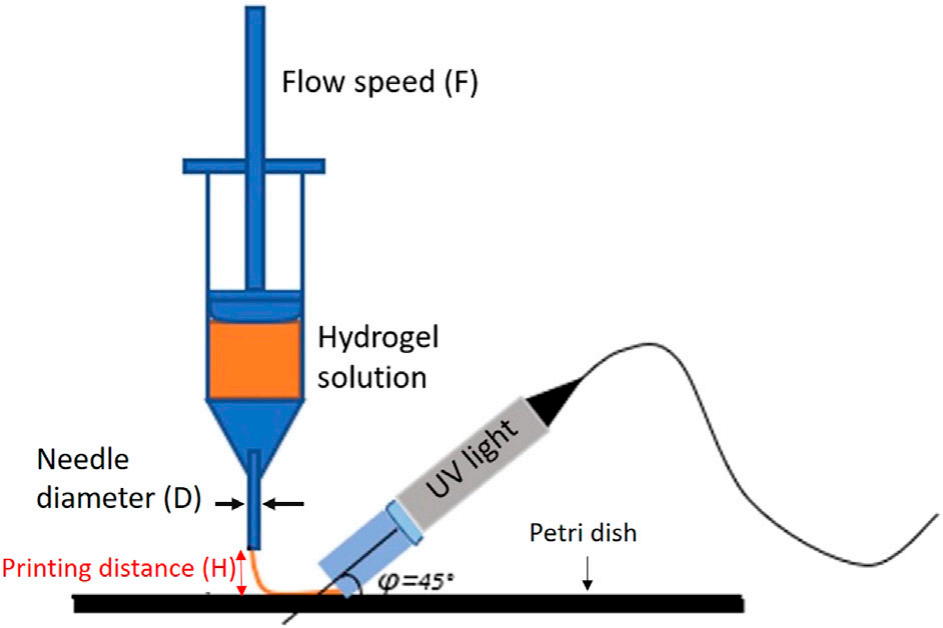
Graphic explanation of the printing system and printing parameters defined.

During the printing process, the distance between the nozzle and the petri dish was kept constant (*H* = 0.24 mm), while the different flow speeds (F) were tested to optimize the printing process (see Figure 1).

### 3.3 Line Printing

The flow speed, F, is the most crucial parameter influencing the printability, due to its impact on the extrusion output. First, it was looked at how the flow speed affected the final shape of the line printing. The printed line width (W) was taken as a representative quality parameter to carry on the resolution of the extruded ink.

### 3.4 Angle Printing

The accuracy of the angle printing was assessed, paying particular attention to acute angles (i.e., angle<90°). The bio-ink’s proclivity for spreading also influences print resolution due to gravity that could be solved by reducing the extrusion rate or speeding up the movement speed. It is essential to consider this aspect to understand how much the diffusion problem affects the print quality, and the layer height error will accumulate layer by layer until the end of the process.

### 3.5 Evaluation of the Printed Lattice

The first step in the evaluation was the measurement of the printed line width using the KEYENCE-VHX microscope (KEYENCE, Japan). The experiments were carried out three times for each sample.

The second step was to assess the printing accuracy of the printed structures by comparing them to the theoretical shape, which was given as an input to the software in the form of stl files. All three solutions were printed in the form of a cube, composed of 2 layers, 0.25 mm thickness, with pores ranging from 5 × 5 mm to 2 × 2 mm, and the diffusion rate was calculated for each printed lattice. To quantify the differences between the theoretical ( $A_{Th}$ ) and the experimental area ( $A_{Re}$ ), the diffusion rate (He et al., 2016) was calculated as follows:

$$\Phi =\;\frac{A_{Th}-A_{Re}}{A_{Th}}\;\times 100\;\%.$$

#### 3.5.1 3D Structures: Multilayers and Tubular Structure

To guarantee the link between the layers and to examine the quality of the process, it is necessary to test multilayer structures. Three different numbers of layers were studied, and each layer had a fixed height of 0.25 mm. For the measurement of the experimental height, a caliper was used. As the first step for the vascular structure, a tubular structure was printed, and the accuracy of the printing sample was examined in terms of shape fidelity.

### 3.6 Tensile Test

To understand the mechanical properties of the bio-ink, tensile tests were performed using the Uniaxial SHIMADZU AUTOGRAPH AGS-X tensile machine with a cross-head speed of 0.02 mm/s. The tensile machine measures the force and the displacement, and load data were acquired and converted into stress and strain values by knowing the dimensions of the samples. Stress and strain were calculated using the following equations:

$$\sigma =\frac{F}{A},$$ (2)

$$\varepsilon =\frac{\Delta l}{l_{0}}*100,$$ (3)

where σ  is the stress, F is the force, A is the area of the sample calculated as  thickness×width , ε refers to the strain, and Δl is the difference between the final and the initial ( $l_{0}$ ) length of the sample. The elasticity, that is, Young’s modulus (YM) of the material, was estimated as the slope of the initial portion of the linear segment of the stress–strain curve between strains 0.05 and 1.5%. The hydrogel solution utilized in the bio-printing procedure was cross-linked using the same UV light and at the same time immersed in the CaCl_2_ solution. The tensile tests were carried out at room temperature on dumbbell-shaped samples. The ASTM D412 standard approved this shape, and it was then scaled down for the tests. Note that the dumbbell shape is required to perform a tensile test with the available equipment since a printed structure will be damaged in the grips. Therefore, the dumbbell shape will be enough as an indicator of the mechanical strength of the bio-inks.

### 3.7 Statistical Analysis

All the experiments were repeated three times. The points and the bars presented in each graph represent the average of three samples, and the error bars present the range of the values. The error in the measurements was considered to have a Gaussian distribution in this study. For the rheological test, *R* ^2^ was calculated to evaluate the fit of the rheological model (Eq. (1)).

## 4 Results

### 4.1 Rheological Test

**FIGURE 2:**
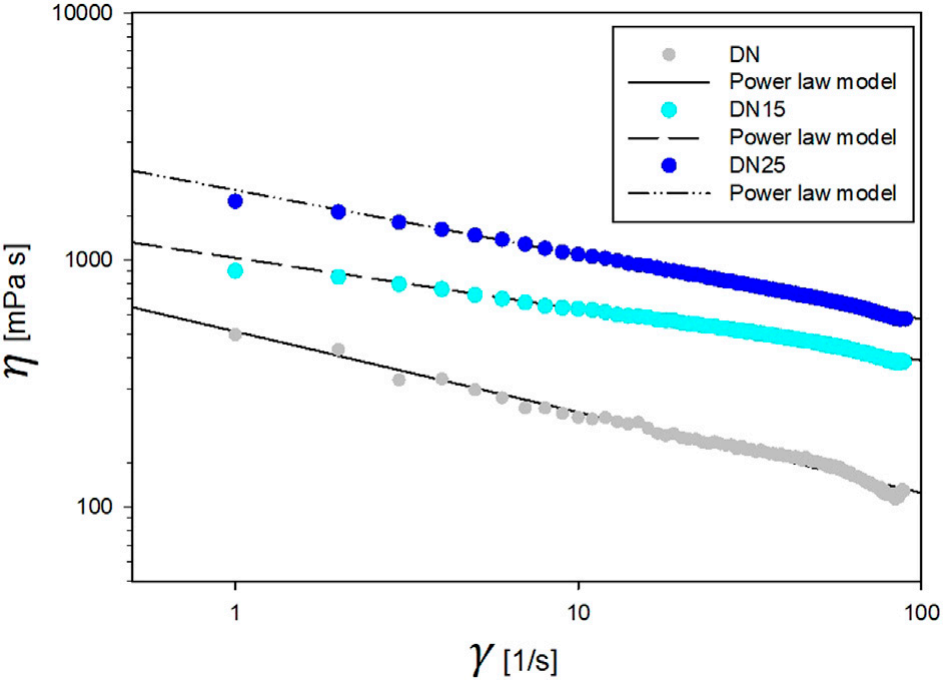
Rheological properties of DN solutions.

As indicated in Figures 2, 4, the increases in the glycerol concentration show an increase in network viscosity. As can be seen, the power-law model can well describe the rheological behavior since for each trend 0.9767 < *R* ^2^ < 0.9931. The model parameters were obtained by non-linear regression at different glycerol concentrations, as reported in Table 2. Rheological results of DN35 are not present in Figure 2 due to the high viscosity of the solution that makes it impossible to test this formulation with the same geometry and settings.

**TABLE 2:** Rheological parameters of DN hydrogel solutions with different glycerol concentrations. Consistency (K) and power law (n) indices were obtained through curve fitting.

Solution	$K\;\left[mPas^{n-1}\right]$	n [−]
DN	428.1923	0.722
DN15	980.833	0.804
DN25	1846.9	0.750

The solutions exhibit a pseudo-plastic shear-thinning flow behavior typical of polymer solutions ( n <1). As the glycerol concentration increased, the solutions maintained the same shear thinning behavior ( n lower than 1) and became thicker ( K increases).

### 4.2 Line Printing

**FIGURE 3:**
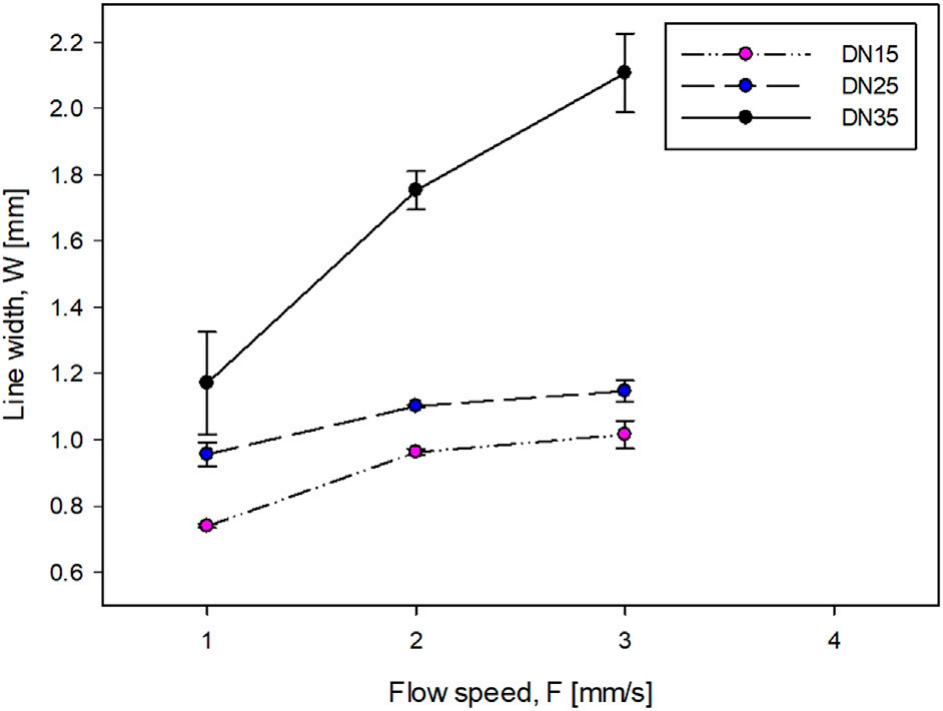
Influence of the flow speed on the line width.

**FIGURE 4:**
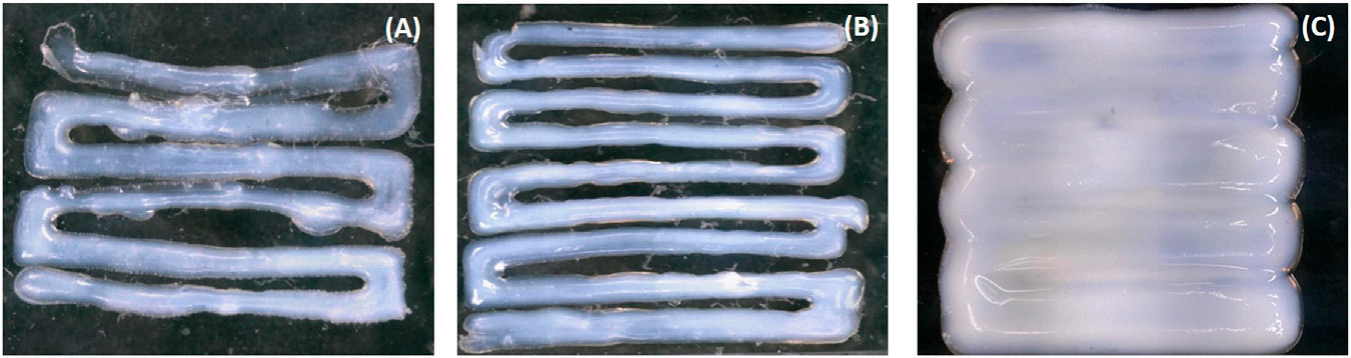
Line printing with optimal flow speed of 2 mm/s: **(A)** DN15, **(B)** DN25, and **(C)** DN35.

In Figures 3,4, the DN is not present because it was impossible to obtain a clear line at any flow speed. This situation makes the DN solution not suitable for this work.

The accuracy of the printing sample is inversely proportional to the line width (Figures 3, 4). For the DN with glycerol (Figure 4), as the flow speed increases, the line width increases. The printing process of the DN35 solution has been challenging due to its high viscosity that was causing the needle occluding and non-continuous filament printing. On the other hand, the printing of the DN15 solution was complex due to the low solution viscosity that was causing the splashing during the printing process. Therefore, the optimal glycerol concentration is 25%.

The optimal flow speed was 2 mm/s. This was defined because it was impossible to obtain a homogeneous shape at lower speeds due to the formation of droplets rather than uniform filaments. On the contrary, at higher speeds, it was impossible to obtain clean lines because the amount of flowing solution was higher than the space between the lines. It should be noted that the minimum line width is greater than the needle diameter (D). This behavior is known as the die-swell phenomenon (or the Barus effect) which occurs at the tip of the needle, and the viscoelastic stresses are canceled within the tip of the needle (Bagley and Duffey, 1970).

### 4.3 Angle Printing

**FIGURE 5:**
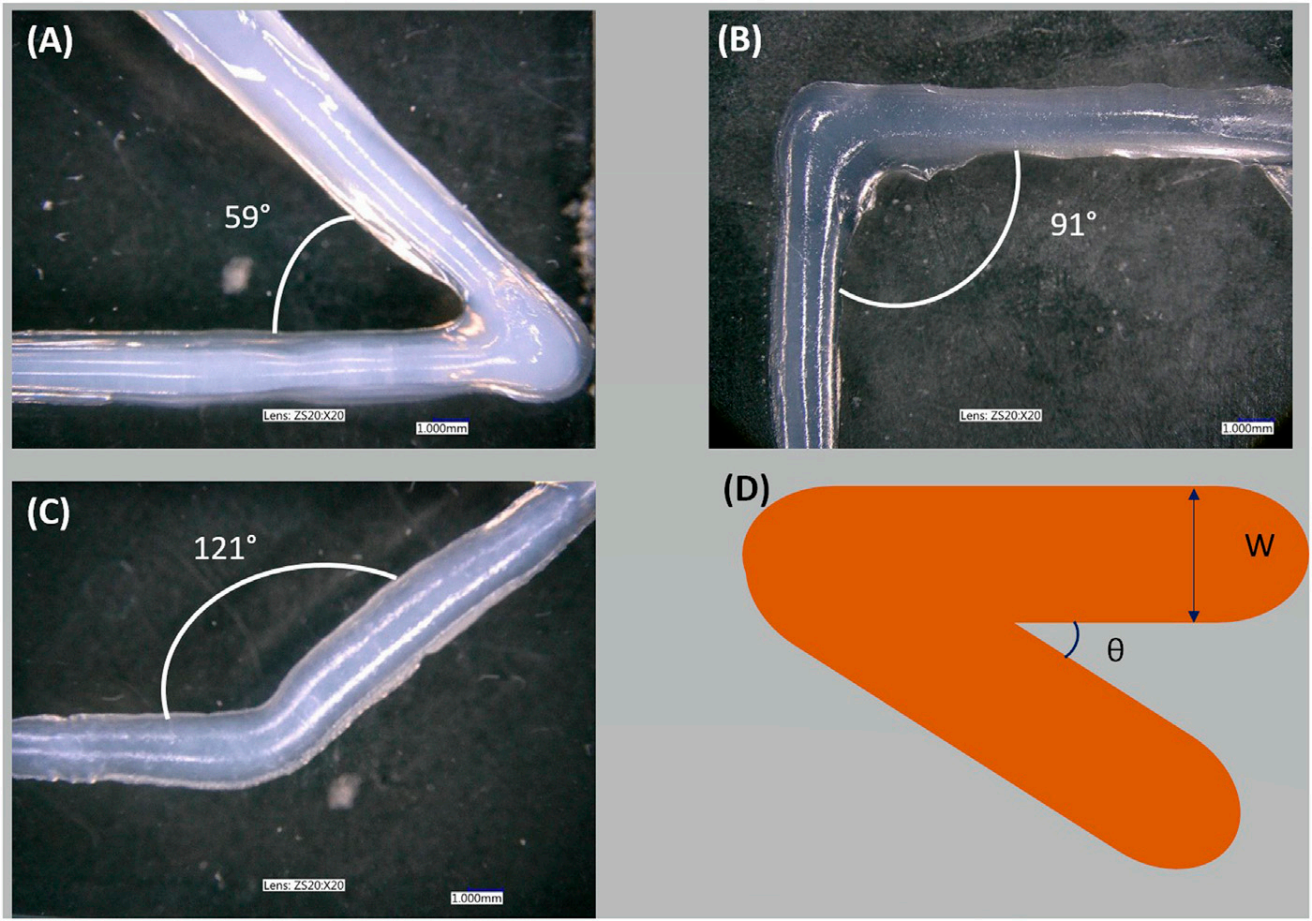
Sharp corner printing: **(A)** shape of acute angle printing, **(B)** shape of right angle printing, **(C)** shape of obtuse angle printing, and **(D)** schema of the overlap.

The quality of the printed angle was measured. Figure 5 shows corners printed with DN25, and Table 3 presents the desired (i.e., reference) angle and the printed (i.e., real) angle. As shown in table 3, the setting parameters and the viscosity of the DN25 formulation resulted in good extrudability, printing accuracy, and the proper shape fidelity.

**TABLE 3:** Comparison between the reference and the printed angle value.

Image	Reference	Real
A	60°	59° ± 1
B	90°	91° ± 1
C	120°	121° ± 1

### 4.4 Lattice Printing

**FIGURE 6:**
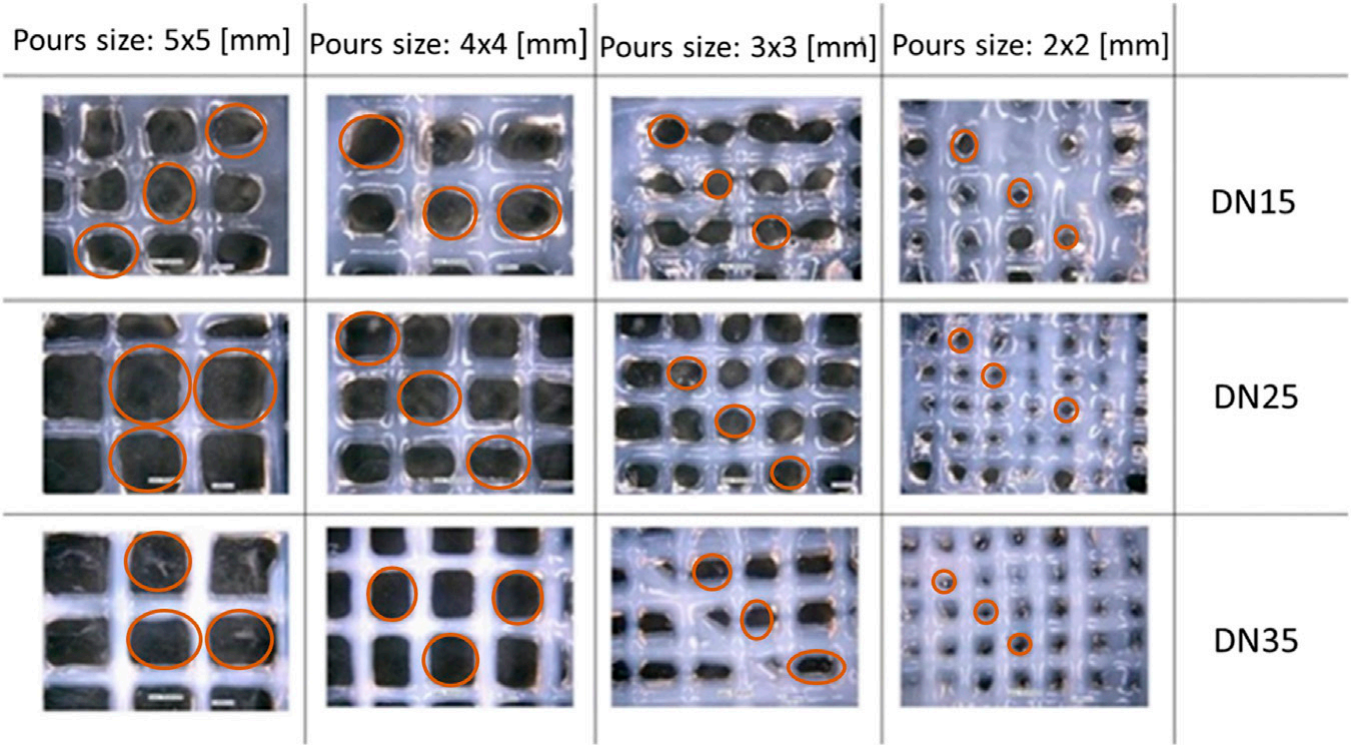
Lattice structure: *D* = 0.41 mm, *H* = 0.240 mm, and *F* = 2 mm/s. Marked in red are the pores that were taken into consideration for the measurements.

**FIGURE 7:**
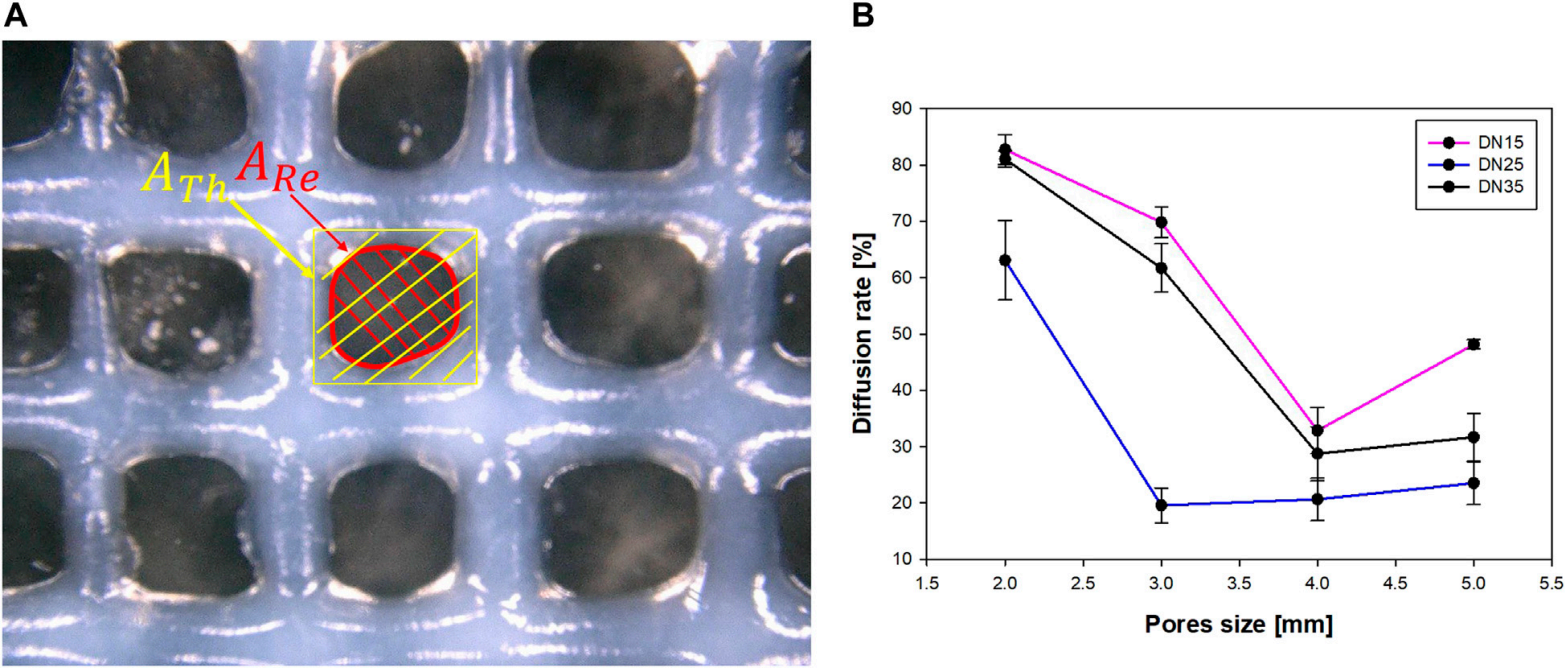
**(A)** Diffusion rate parameters and **(B)** relation between the pore size and diffusion rate.

In Figure 6, microscopic observation showed that the experimental pore area is smaller than the theoretical pore area and tends to deform (as the pore size decreases) from quadratic to circular with irregularities, as shown in Figure 7A.

The parameters that were properly considered for the diffusion rate evaluation are shown in Figure 7A.

The reference area can be well described as a rectangle area, while the real area is described as a circular area. After using the microscope to determine the area, it was observed that as the pore size grows larger, the diffusion rate decreases until the pore size approaches 4 × 4 mm. The overlapped layers diffused due to the gravity influence on the printing process, at the intersection. This phenomenon results in a big diffusion rate, which was most evident in the samples with the smallest pore sizes (2 × 2 mm).

### 4.5 3D Structures: Multilayers and Tubular Structures

**FIGURE 8:**
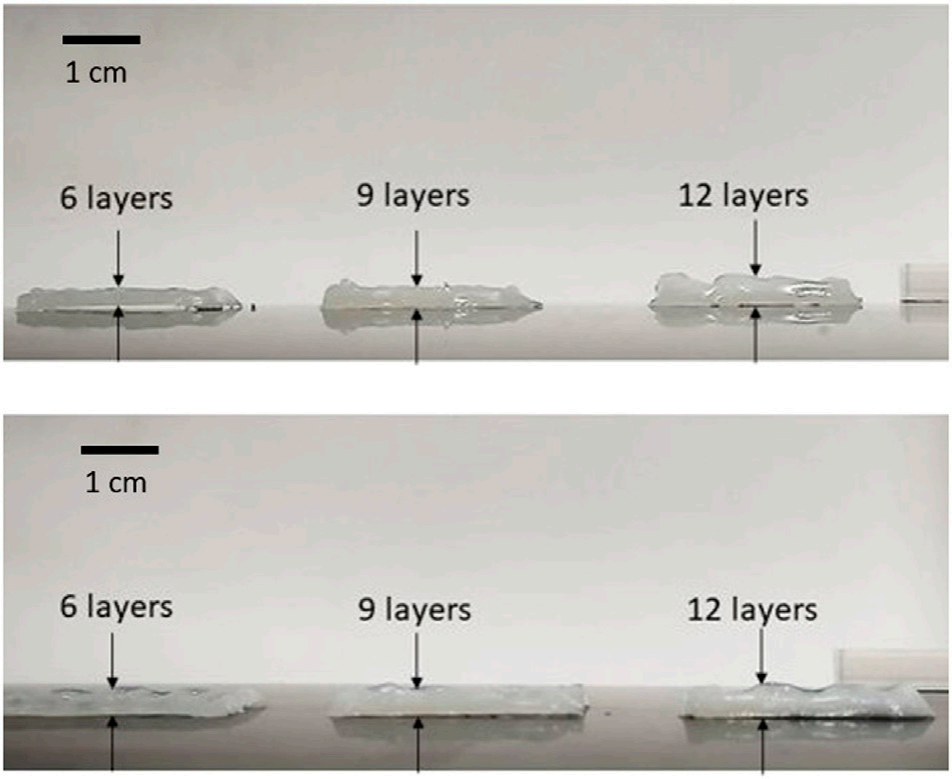
Multilayer structures printed with DN25. *D* = 0.41 mm, *H* = 0.240 mm, and F = 2 mm/s.

**FIGURE 9:**
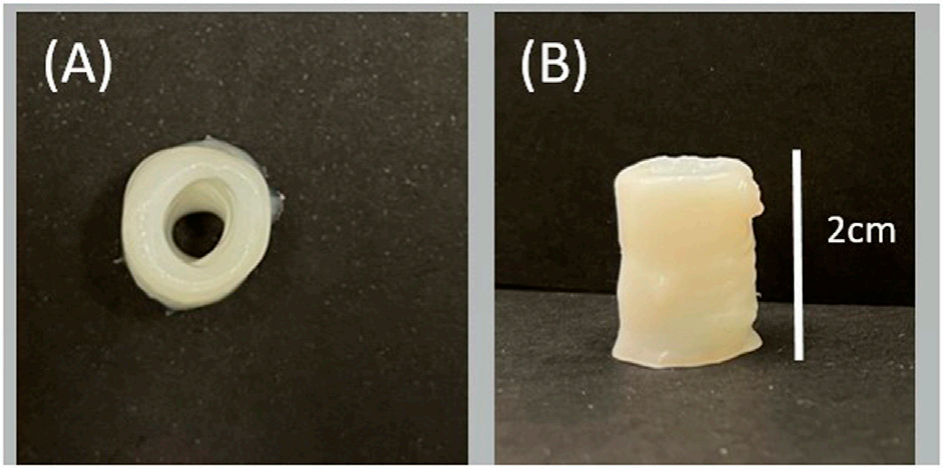
3D tubular structure printed with DN25 using REGEMAT 3D BIO V1: **(A)** top view and **(B)** side view.

Figure 8 shows the results obtained for 3D structures. Due to the material’s high viscosity for the DN35 solution, the needle was constantly occluded during the printing process, making the extrusion procedure unviable. The measurements of the overall height of the printed structure are shown in Table 4. Small diameter tubular structures represent a vascular network’s first step. Thanks to optimizing the ink viscosity and the printer parameters, it is feasible to replicate a tubular structure with control over its dimensions. Figure 9A shows the top view and Figure 9B shows the side view of the tubular structure. The height was 2 cm, and both were printed with the DN25 solution using REGEMAT 3D BIO V1. As for the lattice printing, the diffusion rate was evaluated. In the case of the tubular structure, the average diffusion rate was 4.25%. The reference diameter was 7 mm, and the diameter for the three tested samples was 6.77, 6.87, and 7.05 mm, showing a maximal error of 0.3 mm, which is a sign of a good shape accuracy.

**TABLE 4:** Comparison of desired and experimental heights for the two DN solutions.

Desired height [mm]	Experimental height [mm]	
	DN 15	DN 25
1.5	1.81	1.48
2.25	2.17	2.14
3	2.93	2.70

### 4.6 Tensile Test

**FIGURE 10:**
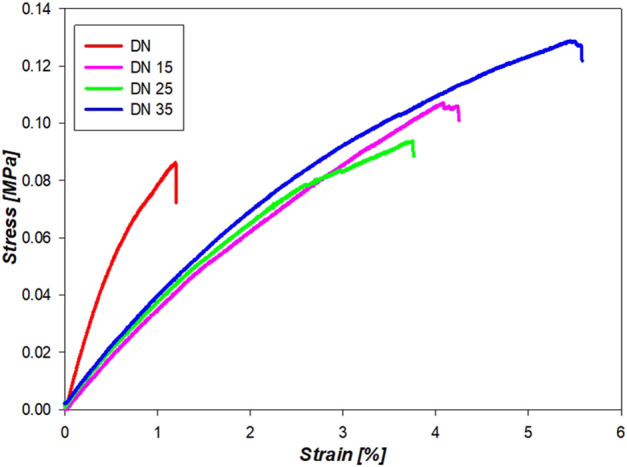
Mechanical properties of the double network solution in the dumbbell shape.

**FIGURE 11:**
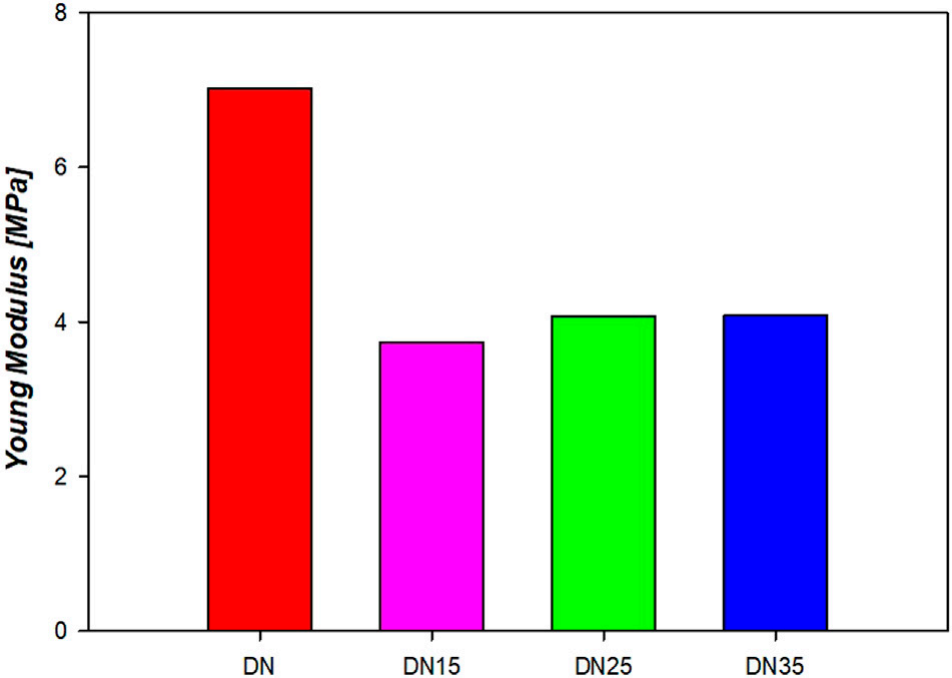
Young’s modulus of the double network hydrogels.

Figure 10 shows the curves that accurately represent the trend, and the average YM is represented in Figure 11. It is possible to see from Figures 10,11 that the presence of glycerol increases the break stress and break strain of the samples. Due to the increase in the glycerol concentration, the elasticity and hence the YM increases. The decrease in the YM on the sample with and without glycerol can be due to the large quantities of glycerol that may reduce the interactions inside the network. In these formulations, a “cross-linker” effect might be present, which decreases the free volume and the molecular mobility of the polymer, thus reducing the flexibility of the hydrogel.

## 5 Discussion

The study of the printability of hydrogels is a journey from one-dimensional to 3D structures (He et al., 2016). Among the 3D prints of the most significant interest for tissue engineering are tubular structures and lattice structures. Tubular structures are globally used in scaffold design, especially for vascular structures, so it is essential to control the printability and shape accuracy of the printing material. Flow speed affects the lattice printing quality. Lattice structures are widely used in every type of cell-laden scaffold, so it is crucial to control the printing quality accurately (Yu et al., 2018).

The experiments presented in this study allowed finding optimized printing parameters, such as flow speed (F) and viscosity, to obtain constructs with high shape fidelity and printing accuracy. It was demonstrated that REGEMAT 3D BIO V1 could be used for 3D bioprinting of a tubular structure. Note that the linearity of a printed structure could be improved if the pressure flow of the printer could be adapted. However, this is one of the limitations of REGEMAT BIO V1, which only allows adapting the flow speed. On the other hand, the focus of this work is on the biomaterial, and despite the fact that the pressure flow cannot be optimized, this study demonstrates that a high level of printing accuracy can be achieved. Concerning the viscoelastic nature of the influence of the solutions on the printing quality, during the bioprinting process, the deposited hydrogel is subjected to deformations, which may generate sagging or collapse. In addition, pore occlusion can occur due to fusion between adjacent filaments after printing. The two main forces underlying these phenomena are gravity, overall structure loss due to compression or sagging, and surface tension, causing filaments to adopt shapes that minimize the surface area (Ribeiro et al., 2020) (He et al., 2016) (Yu et al., 2018). The use of alginates influences the ink’s biocompatibility due to its several desirable qualities for biomaterials (Rowley et al., 1999). In contrast, it contains no cell ligands (Genes et al., 2004) (Alsharabasy et al., 2016); hence, future biocompatible tests will include grafting cell adhesion peptides on alginate to promote cell attachment. The use of PEGDA has a considerable impact on the printing accuracy of the 3D structures, owing to its rapid UV cross-linking capability. As presented in this study, the addition of glycerol enhances the viscosity of the solutions, allowing them to accomplish good printing accuracy. In addition, it makes alginate/glycerol-based networks more appropriate for printing than pure alginate-based networks. Out of those methods used for printing, DN25 was found to be the best. This solution proved to have a viscosity that was easy to extrude and superior shape accuracy than other solutions. A set of appropriate printing process parameters is presented through research into print processing and systematic analysis of the hydrogel structure. Different flow speeds were tested and the optimal was 2 mm/s. Different ink viscosities were evaluated and the optimal was 25% of glycerol. With these settings, good accuracy of the line printing was found. Future studies will further characterize hydrogels embedded with cells, combining good printability and biocompatibility.

## 6 Conclusion

In this study, a series of experiments were conducted to investigate the hydrogel printability in REGEMAT 3D BIO V1 and to fabricate the 3D structure. Since the ink viscosity and the flow speed are the most critical factors influencing printing quality, this study found the double network approach the best solution to bio-print complex 3D structures, such as tubular structures with a commercial bioprinter. With several concentrations of glycerol, the printability of the ink was optimized, and the 25% yielded the greatest results in terms of printing accuracy. The ability to optimize viscosity based on the bioprinter and being cost-effective are the two advantages of this ink. These findings can be used to create multilayered and tubular structures with a high level of shape precision. Even though these results are promising, more work is required to improve tube mechanics and to study the vascular cell response. Furthermore, similar tests in microgravity conditions could be performed to understand the influence of the gravity and optimize the printing process.

## Data Availability

The original contributions presented in the study are included in the article/Supplementary Material; further inquiries can be directed to the corresponding author.
